# The Impact of Sleep Disturbance on Regional Brain Volumes

**DOI:** 10.1093/geroni/igaa057.1521

**Published:** 2020-12-16

**Authors:** Shanna Burke, Tan Li, Adrienne Grudzien, Christopher Barnes, Kevin Hanson, Steven DeKosky

**Affiliations:** 1 Florida International University, Miami, Florida, United States; 2 University of Florida, Gainesville, Florida, United States; 3 University of Florida College of Medicine, Gainesville, Florida, United States

## Abstract

Sleep disruption has been associated with increased beta-amyloid deposition and greater risk for later development of Alzheimer’s disease. Studies indicate that sleep disturbance correlates with regional brain volumes, but data are limited. We sought to determine the effect of sleep disturbance on regional brain volumes by cognitive and apolipoprotein e (APOE) e4 status. We conducted a secondary analysis of the National Alzheimer’s Coordinating Center (NACC) Uniform Data Set using complete structural imaging data from 1,371 participants (mean age: 70.5; SD: 11.7). Multiple linear regression was used to estimate the adjusted effect of sleep disturbance (via Neuropsychiatric Inventory Questionnaire) on regional brain volumes through measurement of 30 structural MRI biomarkers. Sleep disruption was associated with greater volumes in the right and left lateral ventricles and greater volume of total white matter hyperintensities (p<.05). Lower mean volumes in total brain, total gray matter, and total cerebrum grey matter volumes, and in 12 hippocampal, frontal, parietal, and temporal lobe volumes were observed among participants who reported sleep disturbance. Males, Hispanic participants, and those with less education were more likely to report sleep disruption. Cognitive status moderated the relationship between sleep disturbance and lateral ventricular volumes, while APOE e4 moderated the effect between sleep disturbance and parietal lobe volumes. These findings suggest that disrupted sleep is associated with atrophy across multiple brain regions and ventricular hydrocephalus ex vacuo, after controlling for intracranial volume and demographic covariates. The influence of cognition and APOE e4 status indicates that this relationship is affected by co-occurring physiological processes.

